# Cost Effectiveness Analysis and Payment Policy Recommendation—Population-Based Survey with Big Data Methodology for Readmission Prevention of Patients with Paroxysmal Supraventricular Tachycardia treated with Radiofrequency Catheter Ablation

**DOI:** 10.3390/ijerph17072334

**Published:** 2020-03-30

**Authors:** Chien-Lung Chan, Ai-Hsien Adams Li, Hsiang-An Chung, Dinh-Van Phan

**Affiliations:** 1 Department of Information Management, Yuan Ze University, Taoyuan 320, Taiwan; s1056225@mail.yzu.edu.tw (H.-A.C.); dvan2707@due.edu.vn (D.-V.P.); 2 Innovation Center for Big Data and Digital Convergence, Yuan Ze University, Taoyuan 320, Taiwan; 3 Division of Cardiology, Far Eastern Memorial Hospital, Taipei 220, Taiwan; 4 Statistics and Informatics Department, University of Economics, The University of Danang, Danang 550000, Vietnam; 5 Teaching and Research Team for Business Intelligence, University of Economics, The University of Danang, Danang 550000, Vietnam

**Keywords:** cost-effectiveness, paroxysmal supraventricular tachycardia, radiofrequency catheter ablation, big data analytics, DALY

## Abstract

Recurrence of paroxysmal supraventricular tachycardia (PSVT) has been reported to be lower in patients treated with radiofrequency catheter ablation (RFCA) than in those who are not. Few population-based surveys have stated the cost-effectiveness related to this treatment. We, therefore, performed a nationwide retrospective study using National Health Insurance Research Database (NHIRD) data from 2001–2012 in Taiwan. The incidence of PSVT-related admissions was computed from patients’ first admission for a primary PSVT diagnosis. There were 21,086 patients hospitalized due to first-time PSVT, of whom 13,075 underwent RFCA, with 374 recurrences (2.86%). In contrast, 1751 (21.86%) of the remaining 8011 patients who did not receive RFCA, most of whom had financial concerns, experienced PSVT recurrence. The relative PSVT recurrence risk in those who did not receive RFCA was 7.6 times (95% CI: 6.67–8.33) that of those who did undergo RFCA. In conclusion, the PSVT recurrence rate was much higher in patients who did not receive RFCA at their first admission. Furthermore, RFCA proved cost-effective, with the ratio of the incremental cost-effectiveness ratio (ICER) and gross domestic product (GDP) being only 1.15. To prevent readmission and avoid incremental cost, the authority could provide a financial supplement for every patient so that the procedure is performed, reducing the PSVT-recurrence life-years (disease-specific DALY).

## 1. Introduction

Paroxysmal supraventricular tachycardia (PSVT), which is frequently present in medical emergencies, is a common type of rapid arrhythmia. The annual incidence rate per hundred thousand patients has been reported in the US to be around 35, and the prevalence rate per thousand population has been reported to be 2.25. The incidence is proportional to age and women are more likely to suffer PSVT than men [1]. The incidence of PSVT is bound to increase due to the aging society and high prevalence of cardiovascular diseases. The most common symptom is sudden cardiopalmus, and this often occurs with other systemic symptoms, such as fever, dizziness, systemic asthenia, sweating, dyspnea, and chest tightness. During an attack, the heart rate may suddenly go beyond the upper bound of the normal heart rate, usually up to more than 200 beats per minute, and patients might faint or twitch when it becomes more serious.

Radiofrequency catheter ablation (RFCA) is a surgical treatment for patients who suffer from PSVT, particularly in those who experience side effects of medication or insignificant therapeutic results. Surgeons may suggest conducting RFCA as a permanent cure based on the results of individual patient evaluation. According to surgical results in China, the success rate for surgery is up to 98.1% and the recurrence rate after surgery is 5%. For those in whom PSVT recurs, a subsequent RFCA can be conducted. Clinical treatment by surgery results in significantly more positive outcomes and effectively lowers the incidence of complications [2,3]. The symptoms of PSVT may disappear after surgery, significantly improving the condition of the patient.

In a previous study, at the end of a 9-month effectiveness evaluation period, 66% of patients in the catheter ablation group remained free from protocol-defined treatment failure, as compared with 16% of patients treated with antiarrhythmic drug therapy (ADT). The hazard ratio (HR) of catheter ablation to ADT was 0.30 (95% confidence interval [CI]: 0.19–0.47; *p* < 0.001). Major 30-day treatment-related adverse events occurred in 5 of the 57 patients (8.8%) treated with ADT and 5 of the 103 patients (4.9%) treated with catheter ablation. The mean QoL score improved significantly in patients treated with catheter ablation as compared with those treated using ADT at three months (*p* < 0.001); the improvement was maintained during the course of the study [4].

For second-line therapy for paroxysmal AF, ablation was found to be superior to ADT in terms of improving symptoms and QoL. Ablation of long-standing persistent atrial fibrillation was associated with significant recovery of hemodynamics and exercise capacity, which projected into long-term improvement in QoL [5]. RFCA was reported to be associated with significant increases in the physical component summary score (PCS) and mental component summary score (MCS) in AF patients. Patients without AF recurrence after RFCA exhibited greater improvement in the PCS and MCS than patients who had AF recurrence [6]. However, recurrence of PSVT following treatment with RFCA was lower in previous reports than in patients not treated using RFCA. Most importantly, there have been few population-based surveys or reports addressing cost-effectiveness.

This study aimed to address the following research questions based on Taiwan population data.

- What are the incidence rates of “PSVT-related admission” in patients treated with or without RFCA at first admission?
- What is the difference in the risk of PSVT recurrence between patient groups treated with and without RFCA?
- From the perspective of cost-effectiveness, can RFCA really prevent recurrence of PSVT? Can recurrence of the disease be delayed?
- In a first admission for PSVT, what is the incremental cost of hospitalization required in order to increase recurrence-free life by one year?
- Is there any difference in survival between the two groups (RFCA versus non-RFCA)?

## 2. Materials and Methods 

We collected inpatient expenditures by admissions (DD) from the National Health Insurance Research Database (NHIRD) over the period from January 2001 to December 2012. These data were then analyzed to investigate the re-hospitalization of patients due to PSVT after treatment with RFCA. The National Health Insurance system in Taiwan was initiated in 1995 and the insurance coverage rate is currently higher than 99%. In 1998, the National Health Insurance Administration commissioned the National Institute of Health to establish the NHIRD. In 2000, this database was made public to the academic sector for relevant research on the premise that the privacy of citizens and the security of data are assured. Data are classified based on the International Classification of Diseases, 9th Revision, Clinical Modification (ICD-9-CM). In previous literature, the NHIRD was thought to be an effective tool by which to conduct population-based research on cardiovascular diseases [7]. This study was designed to recruit adult subjects aged 20 years and above diagnosed with PSVT (ICD-9-CM code 4270); we compared the relative risk (RR) between those who underwent RFCA and those who did not and analyzed the comorbid factors related to re-hospitalization of PSVT patients. The data protection and permission protocols were approved by the Institutional Review Board (IRB) of Taipei General Hospital, which has been certificated by the Ministry of Health and Welfare, Taiwan (IRB Approval Number: TH-IRB-0015-0003).

**Figure 1:**
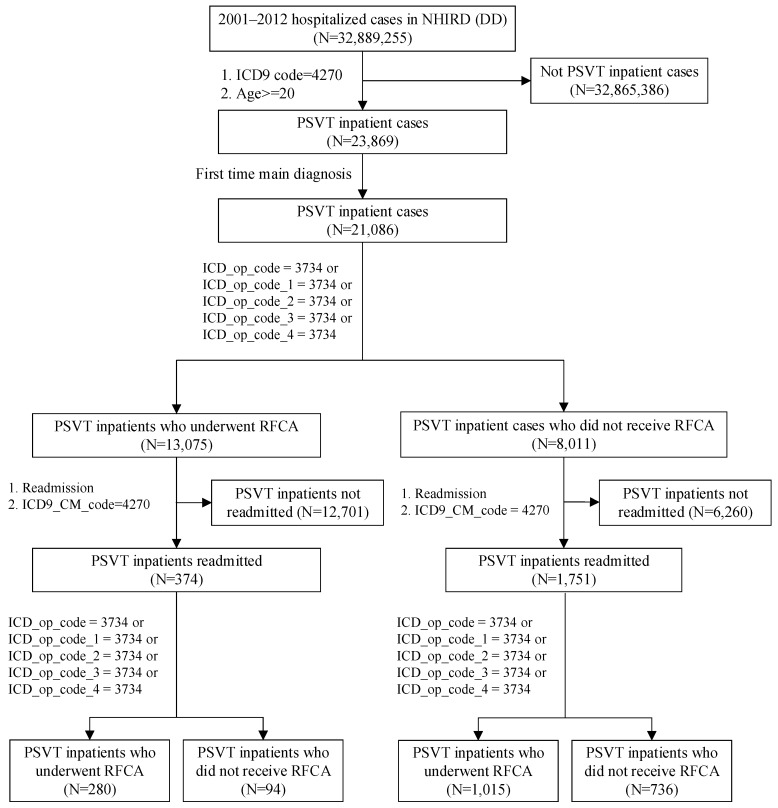
Data-processing flow chart.

Paroxysmal supraventricular tachycardia (PSVT) patient cases (N = 23,869) were selected from the NHIRD in Taiwan (N = 32,889,255) between 2001 and 2012 based on ICD9 code 4270. Among them, there were 21,086 patients diagnosed for the first time, who were classified into two groups: group 1 received RFCA (N = 13,075) based on ICD_op_code = 3734 or ICD_op_code_x = 3734 (x = 1–4); group 2 did not receive RFCA (N = 8011). We then identified patients readmitted (second admission) for PSVT in group 1 (N = 384) and group 2 (N = 1751). Patients readmitted for PSVT were also separated into two groups, those who received RFCA and those who did not (Figure 1).

This study had five assumptions as follows. (1) We regarded the first PSVT-related admission during the study period as the first event in the patient’s lifetime (no PSVT-related admission prior to the study period for any patient). (2) The recurrence of PSVT was PSVT-related readmissions with primary diagnosis ICD9 code 4270. (3) The recurrence of PSVT was also PSVT-related readmissions of patients who received RFCA (with ICD code 4270 as the primary diagnosis). (4) The recurrence of PSVT was PSVT-related readmissions of patients who then underwent re-RFCA after receiving RFCA during the first admission.

We used descriptive statistics to present the characteristics of patients, the number of patients, percentages, means, and SD. Joinpoint Regression Program Ver. 4.7.0.0 was used to calculate the average annual percentage change (AAPC) at alpha = 0.05 [8] and 95% confidence intervals (CI) based on the empirical cumulative distribution function quantile interval (EmpQ) [9]. Relative risk (RR) and odds ratio (OR) analyses were used to compare risk between the two groups. The study used incremental cost-effectiveness ratios (ICERs) [10] to compare the mean lifetime cost and DALY between two groups. Statistical analysis was performed using IBM SPSS Ver. 22.

## 3. Results

Table 1 shows the descriptive statistics for the PSVT patients from 2001 to 2012 in Taiwan. The number of patients diagnosed with PSVT for the first time during hospitalization totaled 21,086. As shown in the table, the age of the PSVT patients ranged from 20 to 102 years (mean, 53 years; SD, 17.7). The proportion of the PSVT patients who were female (55.45%; N = 11,693) was higher than that who were male (44.55%; N = 9394). Analysis of the patients by age strata showed that the greatest percentages of patients were classified into the 20–44 years (32.96%) and 45–59 years (30.82%) age groups.

Another observation was that the PSVT patients usually stayed in hospital for a short period. We classified the patients according to duration of hospitalization, the shortest duration being 0 days (discharged on the same day) and the longest being 753 days (mean, 3; SD, 6.6), into six categories. A hospitalization duration of within one week accounted for the majority of patients (19,874), with the highest proportion of patients falling within the category of 0 to 3 days (16,336; 77.47%) and the second highest in the category of 4 to 7 days (3538; 16.78%). There were 5591 (26.52%) patients hospitalized in spring, which was a little higher than in summer, autumn, and winter (24.96%, 24.42%, 24.42%, respectively).

Table 2 shows the yearly trend in incidence rate in PSVT patients during hospitalization. We used the AAPC to calculate the aggregated measurement of the constant expected time interval, employing the log-linear transition points model. The results showed that the incidence (1/100,000) of hospitalized PSVT patients significantly decreased during the period from 2001 to 2012 in Taiwan, from 24.83 to 20.99 (mean, 22.07) [AAPC, −1.7 (95% CI: −3.0~−0.3)].

**Figure 2:**
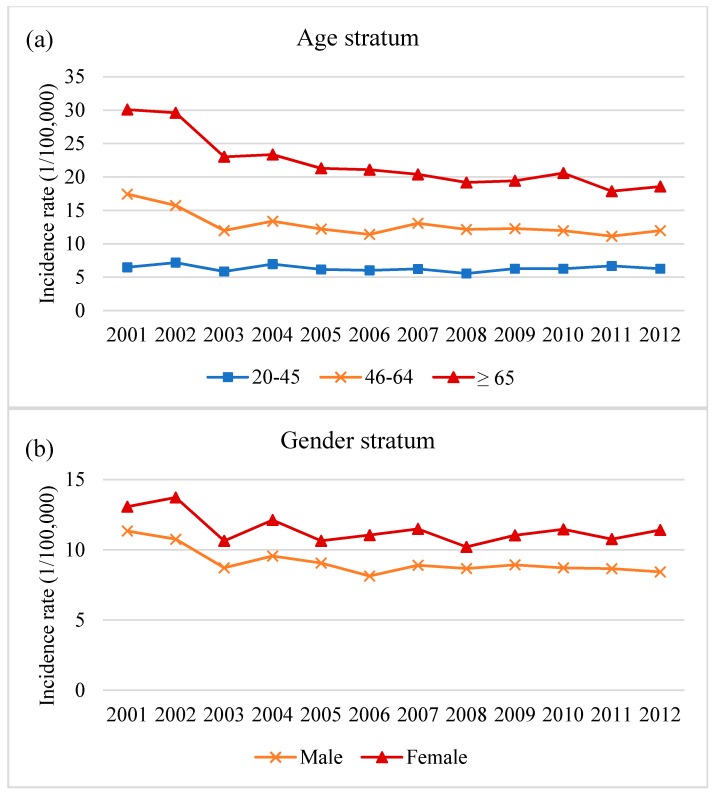
Annual incidence of PSVT-related admissions (1/100,000). (**a**) Annual incidence of PSVT-related admissions of age stratum, (**b**) Annual incidence of PSVT-related admissions of gender stratum.

Figure 2 and Table 3 show the nationwide PSVT morbidity rates in all age and gender categories from 2001 to 2012. The annual population in all age categories was used as the denominator. The annual incidence (1/100,000) in those aged ≥65 was the highest and the trend was a significant decrease, with an AAPC of −4.4 (95% CI: −6.5~−2.5). The trend in the annual incidence was also a significant decrease in the age group of 46–64 years, with an AAPC of −3.8 (95% CI: −6.5~−1.0). The morbidity rate of the female patients was higher than that of the male patients in all age groups, but the annual incidence of PSVT in males significantly decreased more than that in females, with an AAPC of −2.8 (95% CI: −5.0~−0.5) and −1.3 (95% CI: −2.7~0.1), respectively.

**Figure 3:**
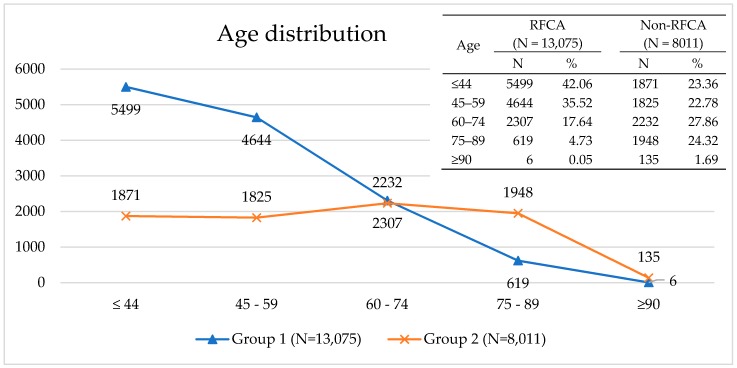
Age at 1^st^ PSVT admission in two groups: group 1 included patients who underwent RFCA; group 2 included patients who did not.

We divided the 21,086 PSVT patients hospitalized between 2001 and 2012 into two groups: group 1 included 13,075 patients who underwent RFCA, while group 2 included 8011 patients who did not. Group 1 accounted for 62.01% of the subjects and group 2 accounted for the remaining 37.99%. The age distribution of group 1 showed a prevalence of patients aged ≤59 years, while the age distribution of group 2 showed an equal distribution across ages. A significant difference was observed between the two groups in that a much higher percentages of patients fell into the age groups of ≤40 and 45–59 years in group 1 than in group 2; while on the contrary, the percentages of patients in the age groups of 75–98 and ≥90 years in group 1 were lower than those in group 2 (Figure 3).

The results shown in Table 4 demonstrate that the decision regarding whether to perform RFCA in PSVT patients resulted in different outcomes in terms of readmission. There were 374 (2.86%) patients re-hospitalized due to PSVT recurrence in group 1 and 1751 (21.86%) in group 2 (7.64 times higher in group 2 than group 1). We applied the Chi-square test to calculate the relative risk (RR) of readmission for PSVT in the patients who underwent RFCA and those who did not. The results showed that the risk of readmission due to PSVT in group 1 (patients who underwent RFCA) was significantly lower than that in group 2, with a RR of 0.13 (95% CI: 0.12–0.15).

The study also analyzed the patients readmitted for PSVT in both group 1 and group 2 who underwent RFCA upon readmission. Among 374 PSVT readmissions in group 1, 280 patients underwent a further RFCA. These patients accounted for 2.14% of the total 13,075 PSVT patients who underwent RFCA at the initial admission, which represented 74.87% of the 374 readmitted PSVT patients who underwent RFCA. In contrast, 1015 patients underwent RFCA upon readmission for PSVT in group 2 (who had no previous RFCA). These readmitted PSVT patients undergoing RFCA accounted for 12.67% of the 8011 PSVT patients who had not previously undergone RFCA, which represented 57.97% of the 1751 PSVT readmitted patients who had not previously undergone RFCA. The odds of performing RFCA again in group 1 was significantly greater than that of performing RFCA for the first time in the patients in group 2 (OR: 2.16, 95% CI: 1.68–2.78) (Table 5).

**Figure 4:**
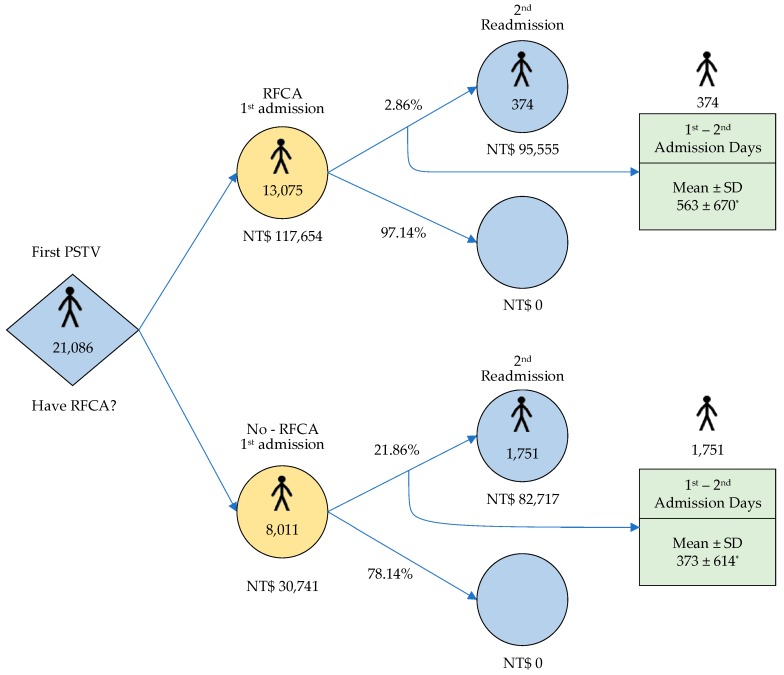
Cost per PSVT patient at the 1^st^ and 2^nd^ admissions with RFCA and without RFCA (* *p*-value < 0.0001).

**Figure 5:**
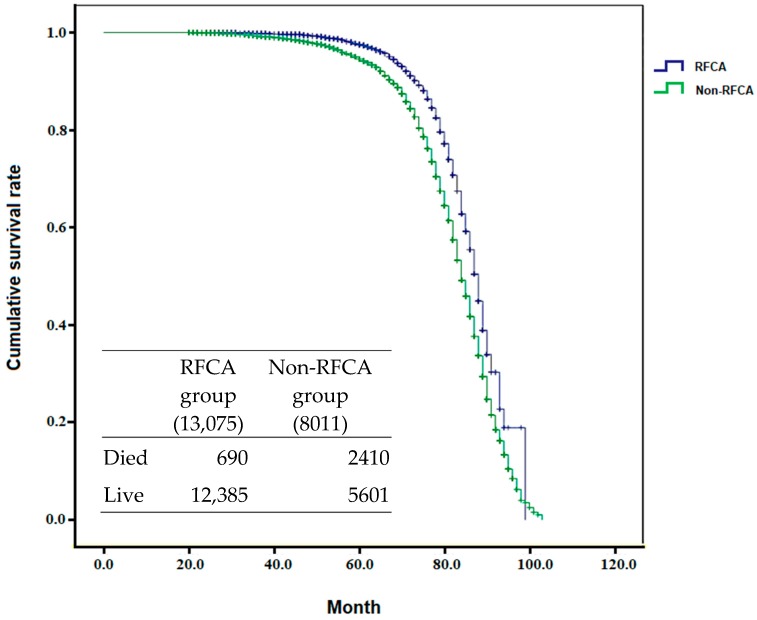
Cumulative survival rate in the two groups (*p* < 0.0001).

The study also analyzed the hospitalization cost for PSVT patients who underwent RFCA from 2001 to 2012 (Figure 4). The expenditure per patient for the first hospitalization in group 1 was higher than that in group 2, at NTD 117,654 versus 30,741 (*p* < 0.001). The cost of readmission in group 1 and group 2 was NTD 95,555 versus 82,717 (*p* = 0.09) per capita. Taking the readmission risk into consideration, the total average hospitalization cost of a 1^st^ admission of a PSVT patient who underwent RFCA was still much higher than that of a PSVT patient who did not, at NTD 120,387 (117,654 + 2.86% * 95,555) compared with NTD 48,823 (30,741 + 21.86% * 82,717) (*p* < 0.05). The DALY was calculated by the sum of years of life lost and years lived with disability for RFCA (155.74) and non-FRCA (1005.94) group. The ICER (NTD 629,411) was calculated using the difference in cost between RFCA group and non-RFCA group (120,387–48,823), divided by the difference in their average effect $\left(\frac{155.74}{13,075}–\frac{1005.94}{8001}\right)$. In consideration of prevention of readmission and the incremental readmission cost between the two groups, the National Health Insurance administration could provide a financial supplement of ICER NTD 629,411 for every PSVT patient in whom RFCA was not performed initially in order to prompt this procedure to be performed and reduce the PSVT recurrence life-years (disease-specific DALY). In addition, the result of comparison between ICER and GDP showed that ICER/GDP was 1.15 times during the period from 2001 to 2012 and the age-corrected cumulative survival rate of patients with RFCA was better than patients who had not undergone RFCA (*p* < 0.0001, Figure 5).

## 4. Discussion

Due to a lack of available information, data prior to January 2001 and after December 2012 were not available. The present study examined the characteristics of PSVT patients from 2001 to 2012 in Taiwan by gender, age, and duration of hospital stay. The trend in the incidence of PSVT was also analyzed using AAPCs in different gender and age categories. In addition, the study examined the recurrence of PSVT in patients who did and did not receive RFCA during the first admission, as well as the medical cost per capita. PSVT occurred most frequently in patients aged ≤59 years, and the proportion of female patients was higher than that of males, similar to a previous study that employed the NHIRD in Taiwan from 2001 to 2010 [11,12]. A study performed in the United States from 1 July 1991 to 30 June 1993 showed that the incidence of PSVT was 35/100,000 person-years [1]. This finding was similar to the present study performed in Taiwan, in which the incidence of PSVT was 24.83/100,000 person-years in 2001, and a decreasing trend was observed from 2001 to 2012.

Although the cost of RFCA was expensive in PSVT patients admitted for the first time, the risk of readmission decreased by around 7.6 times in comparison with patients who did not receive RFCA. RFCA also improves the quality of life of PSVT patients [13]. As shown in Figure 5, the patients who underwent RFCA at first admission for PSVT had a better cumulative survival rate (age-adjusted) than those who did not receive RFCA. In addition, this study only calculated the medical cost, but patients may also have to pay for other costs while living with PSVT. We found that RFCA was performed much less frequently in older patients with PSVT than in younger patients.

Many previous studies have indicated that RFCA is a safe and effective treatment method for PSVT [12,14]. For example, the results of a study of 287 patients who underwent RFCA of a patient population totaling 1250 between April 1992 and December 1998 proved RFCA to be a standard option for PSVT cases [15]. Another study of 454 patients between May 1989 and March 1993 performed at the University of California, San Francisco also proved that RFCA is an effective and safe treatment [16]. RFCA was successfully performed in 56 patients (96.6%), resulting in a significant improvement in quality of life (QoL) at 3 and 12 months after the procedure. There were no major complications related to the procedure. Nine patients (15.5%) had residual arrhythmia, seven of whom underwent repeated ablation, with successful results. The procedure also reduced health-care resource utilization and cost. RFCA proved to be a safe and effective treatment for premature ventricular contractions (PVCs) and a viable alternative to drugs in the presence of disabling symptoms [17]. Our population-based study also indicated a lower risk of readmission for PSVT if the patient had received RFCA at the first admission, and the cumulative survival rate of PSVT patients who underwent RFCA was also improved.

The cost of RFCA is very high and, therefore, countries make decisions as to how to support patients depending on cost-effectiveness ratios. The common threshold is three times the per capita GDP per DALY [18,19]. In fact, this study indicated that the ICER (NTD 629,411) was less than three times (ICER/GDP per capita = 1.15) the average GDP per capita (NTD 547,444) in Taiwan from 2001 to 2012. This finding may be important for the government in terms of policy-making in Taiwan.

The data in this study included re-hospitalized PSVT patients from 2001 to 2012 retrieved from the NHIRD published by the National Health Institute. The assumption of a first admission for PSVT was not based on real occurrence, only hospitalization; however, whether or not the admission date of the first hospitalization was the exact date of PSVT occurrence, the real recurrence was defined by the content of the data.

## 5. Conclusions

This study showed the decreasing trend in the occurrence of PSVT in Taiwan from 2001 to 2012, with an average incidence of 22 per 100,000 patient-years, and the older the patients, the more the decrease. Among the total patient population, female patients and elderly patients accounted for higher proportions than other groups. RFCA reduced the risk of readmission and increased potential survival in the PSVT patients. Considering the 2017 Taiwan GDP per capita, the study recommends that RFCA be performed during the 1^st^ admission for PSVT to reduce the DALY. The study was based on a number of assumptions, including that all patients had no PSVT prior to the study period; that PSVT-related readmissions were likely attributed to recurrence of PSVT; and that re-RFCA after RFCA during the initial PVST admission was based on the primary diagnosis and likely attributed to the recurrence of PSVT. The potential survival benefits and effects of co-morbidities will be considered in the next study.

## Figures and Tables

**Table 1:** Characteristics of 21,086 patients residing in Taiwan diagnosed with PSVT from 2001 to 2012.

Characteristics	PSVT (N = 21,086)	%	Average ± SD	Min	Max
Gender					
Male	9393	44.55%			
Female	11,693	55.45%			
Age (years)			53.2 ± 17.7	20	102
≤44	6950	32.96%			
45–59	6498	30.82%			
60–74	4660	22.10%			
75–89	2774	13.16%			
≥90	204	0.97%			
Hospitalization duration (days)			3.3 ± 6.6	0	753
0–3	16,336	77.47%			
4–7	3538	16.78%			
8–14	890	4.22%			
15–21	184	0.87%			
22–29	66	0.31%			
>30	72	0.34%			
Season					
Spring	5591	26.52%			
Summer	5264	24.96%			
Autumn	5149	24.42%			
Winter	5082	24.10%			

**Table 2:** Annual incidence of PSVT-related admissions (1/100,000).

Year	2001	2002	2003	2004	2005	2006	2007	2008	2009	2010	2011	2012
PSVT first admission	24.83	24.65	21.91	22.9	21.77	21.37	21.85	20.97	21.38	21.37	20.88	20.99
AAPC ^1^	−1.7 (95% CI ^2^: −3.0~−0.3)											

^1^ AAPC = average annual percent change; ^2^ confidence interval.

**Table 3:** Annual incidence of PSVT-related admission by age (1/100,000).

Year	2001	2002	2003	2004	2005	2006	2007	2008	2009	2010	2011	2012	AAPC ^1^ (95% CI ^2^)
Age stratum (years)													
20–45	6.475	7.175	5.846	6.952	6.158	6.018	6.235	5.545	6.273	6.257	6.682	6.260	−0.5 (−1.8~0.9)
46–64	17.434	15.734	11.987	13.375	12.212	11.399	13.070	12.142	12.271	11.954	11.134	11.963	−3.8 (−6.5~−1.0)
≥65	30.092	29.616	23.015	23.359	21.295	21.093	20.388	19.177	19.424	20.584	17.862	18.563	−4.4 (−6.5~−2.5)
Gender stratum													
Male	11.343	10.757	8.725	9.560	9.065	8.136	8.901	8.670	8.933	8.720	8.665	8.428	−2.8 (−5.0~−0.5)
Female	13.069	13.730	10.638	12.124	10.644	11.054	11.494	10.208	11.033	11.460	10.764	11.414	−1.3 (−2.7~0.1)

^1^ AAPC = average annual percent change; ^2^ confidence interval.

**Table 4:** Relative risk of PSVT-related readmission in patients who underwent RFCA and those who did not.

PSVT First Admission N = 21,086	Group 1	Group 2	Relative Risk RR (95% CI)
	With RFCA N = 13,075 (100%)	Without RFCA N = 8011 (100%)	
PSVT readmission	374 (2.86)	1751 (21.86)	0.13 (0.12–0.15)
PSVT no readmission	12,701 (97.14)	6260 (78.14)	

**Table 5:** Odds ratio for RFCA in readmitted PSVT patients who did or did not undergo RFCA at first admission.

	Group 1 (N = 13,075)	Group 2 (N = 8011)	Odds Ratio (95% CI)
	PSVT Readmissions 374 (100%)	PSVT Readmissions 1751 (100%)	
Performed RFCA	280 (74.87)	1015 (57.97)	2.16 (1.68–2.78)
Did not perform RFCA	94 (25.13)	736 (42.03)	
